# A case report of neoadjuvant targeted therapy in stage IIIA non-small cell lung cancer with *BRAF V600E* mutation

**DOI:** 10.3389/fmed.2025.1664182

**Published:** 2025-12-17

**Authors:** Wenying Peng, Susu Qu, Lijuan Cai, Runxiang Yang, Mengyuan Dong, Xiang Li, Fengming Ran, Chunxiang Luo

**Affiliations:** 1 The Second Department of Oncology, The Third Affiliated Hospital of Kunming Medical University & Yunnan Cancer Hospital & Peking University Cancer Hospital Yunnan, Kunming, China; 2 The Second Department of Internal Medicine, Kunming Xishan District People's Hospital, Kunming, China; 3 Department of Pathology, The Third Affiliated Hospital of Kunming Medical University & Yunnan Cancer Hospital & Peking University Cancer Hospital Yunnan, Kunming, China

**Keywords:** *BRAF V600E* mutation, dabrafenib, trametinib, NSCLC, neoadjuvant therapy

## Abstract

For advanced or metastatic non-small cell lung cancer (NSCLC) with actionable gene mutations, first-line targeted therapy significantly prolongs survival compared to chemotherapy and immunotherapy. Perioperative targeted therapy has achieved significant improvement in early- and locally advanced-stage NSCLC patients with epidermal growth factor receptor (*EGFR*) and anaplastic lymphoma kinase (*ALK*) mutations. However, there have been few reports of neoadjuvant treatment in patients with non-small cell lung cancer with rare mutations, such as BRAF (V-Raf murine sarcoma viral oncogene homolog B) V600E mutation. In this report, we present a case of inoperable stage IIIA lung adenocarcinoma with a *BRAF V600E* mutation that underwent radical lung cancer surgery following neoadjuvant targeted therapy. The postoperative pathology review revealed a pathological complete response (pCR). This case illustrates that BRAF and mitogen-activated extracellular signal-regulated kinase (MEK) inhibitor therapy may represent a viable option for neoadjuvant therapy in locally advanced *BRAF V600E* mutant NSCLC.

## Introduction

*BRAF* gene mutations are rare in NSCLC, occurring in approximately 1–5% of patients (1). These mutations are classified into three functional classes based on different signaling pathway mechanisms and kinase activities (2). Among them, the *BRAF V600E* mutation is the most common variant in the V600 subgroup, accounting for approximately 90% of cases. Patients with *BRAF V600E* mutations typically present with adenocarcinoma and are more likely to have multiple metastases at the time of diagnosis. Metastatic or advanced NSCLC patients with *BRAF V600* mutations generally have a poor prognosis and shorter overall survival, with limited effectiveness of standard chemotherapy and immunotherapy regimens (3, 4). *BRAF,* located downstream of the RAS gene, is the most important transduction factor of the MAPK (RAS–RAF–MEK–ERK) signaling pathway and exhibits the strongest kinase activity among the RAF family. Once activated, BRAF sequentially phosphorylates MEK and extracellular signal-regulated kinase (ERK), driving uncontrolled cellular proliferation (5). BRAF inhibitors (e.g., dabrafenib and vemurafenib) selectively occupy the ATP-binding pocket of *BRAF*-mutant monomers, thereby abrogating phosphorylation-dependent activation of the downstream MEK–ERK axis and suppressing tumor cell proliferation (6). Co-inhibition of BRAF and its downstream target MEK has been shown to synergistically heighten anti-tumor efficacy and forestall drug resistance (7–10). The BRF113928 study revealed that patients with advanced-stage NSCLC with *BRAF V600E* mutation had improved prognosis through dual-targeted therapy (11). In NSCLC patients with *BRAF V600E* mutation, the objective response rate (ORR) of dabrafenib combined with trametinib exceeds 60%, regardless of prior treatment status (treatment-naive or resistant to first-line chemotherapy). The median progression-free survival (mPFS) for first-line treatment was 14.6 months, and the median overall survival (mOS) was 24.6 months (12). Therefore, a majority of authoritative guidelines, such as National Comprehensive Cancer Network (NCCN), European Society for Medical Oncology (ESMO), and Chinese Society of Clinical Oncology (CSCO), recommend dabrafenib in combination with trametinib as the first-line treatment for patients with advanced or metastatic NSCLC with *BRAF V600E* mutation (13–15). In addition, the BRAF inhibitors in combination with MEK inhibitors have been established as the standard adjuvant and neoadjuvant therapy for malignant melanoma with *BRAF V600E* mutations based on high-level clinical evidence (16, 17). Targeted therapy in NSCLC is primarily recommended for patients with advanced-stage disease. Evidence supporting the use of BRAF and MEK inhibitors in the neoadjuvant and adjuvant settings for NSCLC remains limited. In this study, a patient with inoperable stage IIIA lung adenocarcinoma and a *BRAF V600E* mutation achieved a partial response (PR) following neoadjuvant targeted therapy. Subsequently, the patient underwent radical lung cancer resection and achieved pCR, suggesting that BRAF and MEK inhibitors may offer the potential for a curative approach in these patients.

## Case presentation

We present the case of a 68-year-old woman who was admitted to Yunnan Cancer Hospital with a 20-day history of persistent cough accompanied by expectoration and mild right chest discomfort. She denied symptoms of hemoptysis, fever, night sweats, or dyspnea, and her Eastern Cooperative Oncology Group (ECOG) performance status was 1. On 24 December 2021, a computed tomography (CT) scan revealed a 4.6 cm × 3.8 cm lesion in the lower lobe of the right lung, as well as significantly enlarged group seven lymph nodes that were observed with a maximum dimension measuring 4.8 cm × 2.7 cm. The patient has a medical history of type 2 diabetes mellitus but has no history of smoking or coal mine exposure. Physical examination revealed features of chronic illness, slightly coarse breath sounds in the right lung, and no palpable enlargement of superficial lymph nodes. Tumor markers were high, with CA125 at 77.30 kU/L and CA15-3 at 41.9 kU/L. On 6 January 2022, a CT-guided biopsy of the right lower lung lesion was performed for pathological evaluation. The immunohistochemistry pathology report issued on 11 January 2022 confirmed the diagnosis of adenocarcinoma. Genetic testing for 11 actionable driver genes, including *EGFR, ALK, ROS1, RET, HER-2, BRAF, KRAS, c-MET,* and *NTRK1/2/3*, using the Amplification Refractory Mutation System Polymerase Chain Reaction (ARMS-PCR) identified a *BRAF V600E* mutation (1799T>A, p.Val600Glu). Additionally, the patient exhibits wild-type results for *EGFR, ALK, ROS-1, RET, HER-2, KRAS, c-MET,* and *NTRK1/2/3* genes. The current diagnosis indicates adenocarcinoma in the lower lobe of the right lung with metastasis to the right hilar and mediastinal lymph nodes (cT2bN2M0), characterized by the presence of the *BRAF V600E* mutation in stage IIIA according to the American Joint Committee on Cancer (AJCC) 8th edition staging system.

**Figure 1:**
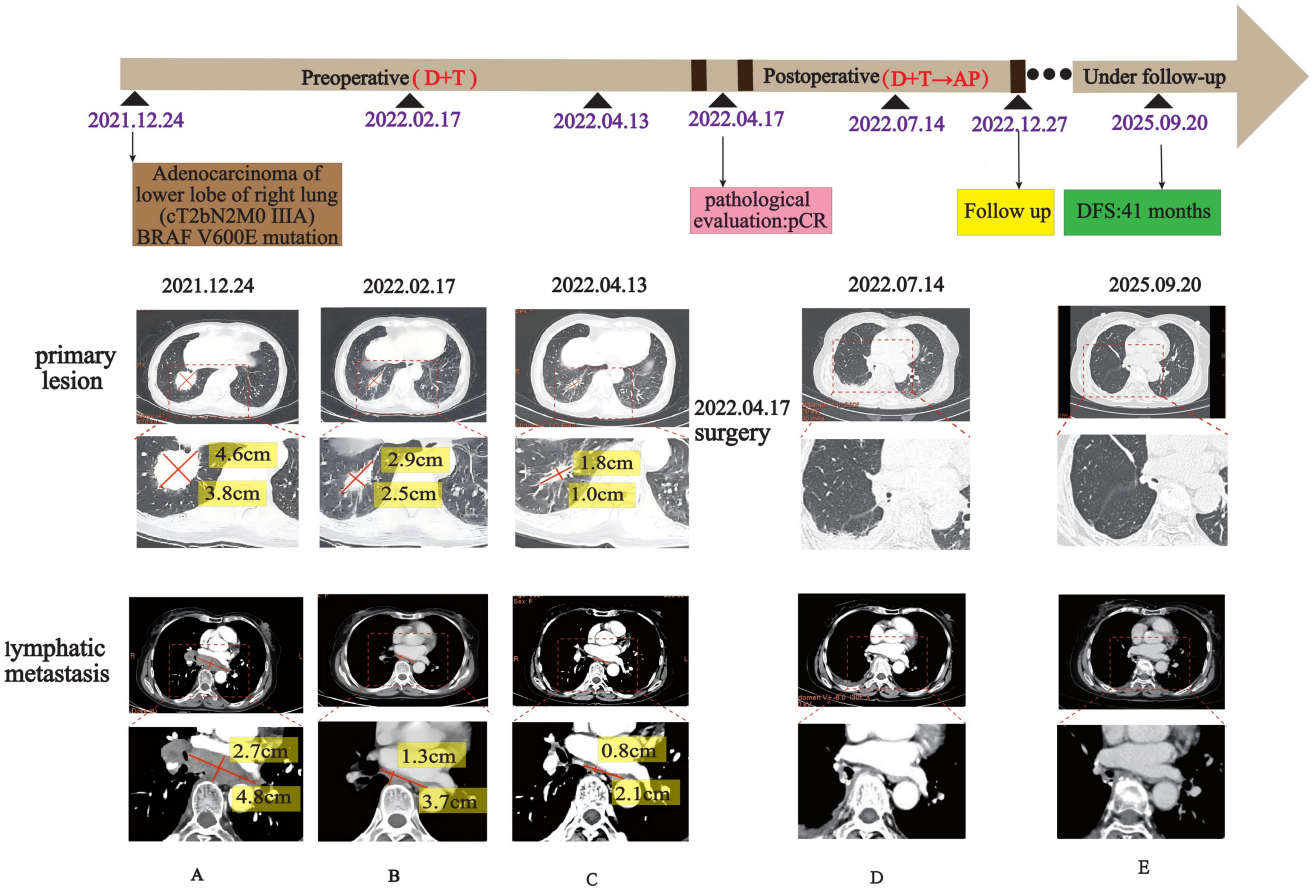
Brief summary of the treatment process. **(A–C)** The changes of chest CT after the adjuvant D+T regimen showed that the lesions in the lower lobe of the right lung were gradually shrunk, and the multiple mediastinal lymph nodes were significantly reduced. **(D,E)** CT review after the operation showed no evidence of recurrence during 41 months of postoperative follow-up. D+T, dabrafenib and trametinib; AP, pemetrexed disodium and carboplatin; DFS, disease-free survival; pCR, pathological complete response.

**Figure 2:**
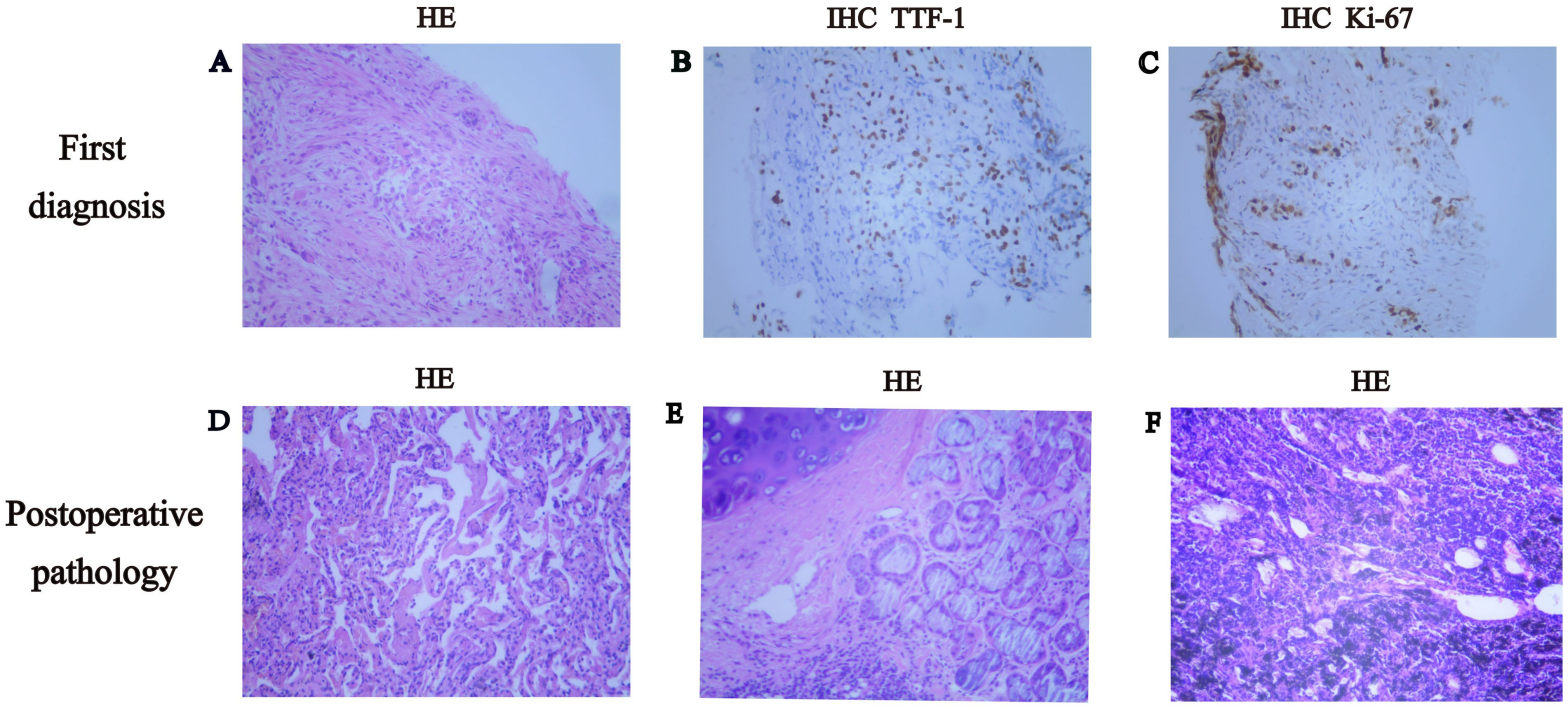
Histopathological assessment of tumor regression. **(A–C)** Pathological images at diagnosis (×100). **(A)** Hematoxylin and eosin (HE) stain of lung adenocarcinoma, **(B)** expression of thyroid transcription factor-1 (TTF-1). **(C)** Expression of proliferation cell nuclear antigen (Ki67). **(D–F)** Postoperative pathological images after treatment with dabrafenib combined with trametinib (×100). **(D)** Postoperative pathological images after 3 months of treatment with dabrafenib combined with trametinib showed tumor remission and normal alveolar epithelium. **(E)** Negative bronchial margin. **(F)** No lymph node metastasis.

After being fully informed of the cost and the subsequent treatment process, the patient carefully considered her options and finally chose targeted therapy. In January 2022, the patient commenced treatment with dabrafenib (Novartis Pharma AG, Basel, Switzerland; 150 mg orally twice daily) combined with trametinib (Novartis Pharma AG, Basel, Switzerland; 2 mg orally once daily). The initial efficacy assessment following more than 1 month of targeted therapy indicated a PR (Figure 1), with the tumor exhibiting a 42.5% reduction according to RECIST 1.1 criteria. After more than 3 months of targeted therapy, the efficacy evaluation according to RECIST 1.1 indicated that the tumor size had reduced by 75.3%. During the medication period, the patient experienced intermittent grade 1–2 fever and cutaneous panniculitis, according to Common Terminology Criteria for Adverse Events (CTCAE) 5.0. On 13 April 2022, a CT scan revealed that the lesion in the posterior basal segment of the right inferior lobe measured 1.8 cm × 1.0 cm (originally 4.6 cm × 3.8 cm), and the diameter of the multiple lymph nodes in the right hilum and mediastinum was less than 1.0 cm (originally group 7: 4.8 cm × 2.7 cm). Considering the significant reduction in both the primary lung lesion and lymph node metastasis, thoracoscopic-assisted radical resection of the right inferior lobe carcinoma and bronchoplasty were performed on 17 April 2022. The final pathology report revealed no tumor cells in the postoperative specimen, and there were no metastatic cancer cells in the lymph nodes or visceral pleura. The pathological evaluation indicated a pCR (Figure 2). The patient received a systemic examination 3 months after the surgery, and no sign of recurrence was observed. The patient received one additional cycle of dabrafenib and trametinib. Subsequently, the patient underwent five cycles of adjuvant therapy with the AP regimen, consisting of pemetrexed 710 mg on day 1 and carboplatin 0.4 g on day 1 of each 3-week cycle. Currently, after 41 months of follow-up post-surgery, the patient remains free of tumor recurrence (Figure 1).

## Discussion

The *BRAF* gene mutation plays a pivotal role in tumorigenesis, tumor development, and prognosis across various cancer types, including malignant melanoma, colon cancer, NSCLC, thyroid papillary carcinoma, and serous ovarian cancer. The prevalence of *BRAF* mutations in NSCLC patients is notably lower (approximately 1–5%) compared to malignant melanoma, with almost half of these patients presenting the *BRAF V600E* mutation (18–20). For NSCLC patients with wild-type *EGFR*, *ALK*, and *ROS1* genes, the prevalence of *BRAF V600E* mutations is approximately 2.2–10.8% due to the mutually exclusive nature of these driver genes (21, 22). At present, dabrafenib combined with trametinib has received Food and Drug Administration (FDA) approval for several indications, encompassing first-line treatment of *BRAF V600E* metastatic NSCLC, postoperative adjuvant treatment of *BRAF V600E*-mutated unresectable melanoma or metastatic melanoma harboring *BRAF V600E* mutation, locally advanced or metastatic anaplastic thyroid cancer (ATC) with *BRAF V600E* mutation, and children aged ≥1 year diagnosed with low-grade glioma (LGG) with *BRAF V600E* mutation. BRAF inhibitors combined with MEK inhibitors have shown promising potential as neoadjuvant therapy in other tumors, such as locally advanced melanoma or metastatic thyroid cancer; however, the evidence in patients with NSCLC remains very limited (23, 24). In this study, we present a case of an inoperable stage IIIA *BRAF V600E* mutation NSCLC patient who received perioperative targeted therapy with dabrafenib and trametinib, subsequently underwent surgery, and achieved pCR. This case suggests that perioperative dabrafenib and trametinib targeted therapy in NSCLC may expedite tumor shrinkage, facilitate stage reduction, enable radical operation, and improve pathological remission rate and survival.

NSCLC patients face a heightened risk of recurrence during the perioperative period, with adjuvant or neoadjuvant targeting/immunotherapy combined with chemotherapy offering potential improvement of disease-free survival (DFS) and quality of life. The efficacy of adjuvant targeted therapy in *ALK* fusion and *EGFR* mutation NSCLC has been demonstrated, while the effectiveness of perioperative targeted therapy for *BRAF V600E* NSCLC remains uncertain. The ongoing NAUTIKA1 (NCT04302025) is a Phase II umbrella trial investigating the efficacy and safety of targeted therapy as adjuvant or neoadjuvant treatment in patients with resectable NSCLC harboring gene alterations, including *ALK, ROS1, NTRK, BRAF V600,* and *RET.* At the 2023 World Congress of Lung Cancer (WCLC), the results indicated the potential efficacy of ALK-TKI in the neoadjuvant treatment of *ALK* fusion NSCLC. The major pathologic response was 66.7%, with three of nine eligible patients achieving pCR and eight patients achieving R0 resection (25). However, clinical trials on adjuvant or neoadjuvant therapy for other rare driver mutations (including *BRAF V600*) in NSCLC have not yet been reported.

As for stage IIIA *BRAF V600E* patients, targeted therapy has shown potential efficacy in neoadjuvant treatment in a few cases. Liu et al. (26) described a case of cT1cN2M0 NSCLC with programmed cell death-ligand 1 (PD-L1) expression of 90%, in which the patient received 2 months of dabrafenib plus trametinib before undergoing complete resection and exhibiting major pathological response (MPR). Adjuvant dabrafenib/trametinib was given for one cycle, but the follow-up time reported was only 3 months. Huang et al. (27) subsequently described the case of a 61-year-old patient with stage IIIA (cT2N2M0) NSCLC harboring *BRAF V600E* and *SETD2* co-mutations, PD-L1 expression of 80%, microsatellite stable status (MSS), and a tumor mutational burden (TMB) of 1 mut/Mb. The patient achieved a partial response after identical neoadjuvant therapy, and subsequent surgery demonstrated pCR. Although no further adjuvant treatment was administered, the patient remained disease-free at 8 months. In this study, we present a cT2bN2M0 *BRAF V600E* mutant NSCLC case that was initially inoperable due to N2 disease and a higher T stage than previously reported cases but achieved PR following 3 months of dabrafenib and trametinib. Right upper lobectomy with systematic mediastinal lymphadenectomy revealed pCR. Adjuvant management comprised one cycle of dabrafenib and trametinib, followed by five cycles of cisplatin–pemetrexed chemotherapy. To date, the patient has remained recurrence-free for more than 41 months, representing the longest disease-free survival yet documented for neoadjuvant targeted therapy in this molecular subset. These cases show that targeted neoadjuvant treatment strategy results in rapid tumor regression, pathological remission, and reduced stage in patients with potentially resectable stage IIIA *BRAF V600E* tumors. However, clinical evidence remains limited, currently restricted to just a few case reports. The efficacy and safety of neoadjuvant targeted therapy in this population should be further evaluated in large, multicenter, randomized controlled studies in the future.

Dudnik et al. (28) reported that all patients (*n* = 39) with BRAF-mutated advanced non-small cell lung cancer were MSS- and PD-L1-positive. Additionally, 2 of the 39 tumors exhibited high TMB, whereas 37 had low or intermediate TMB. PD-L1 expression has been associated with improved outcomes in patients with unresectable stage IIIA NSCLC receiving the consolidation therapy with durvalumab following chemoradiotherapy (the PACIFIC regimen). However, the predictive relevance of PD-L1 expression for targeted therapy in *BRAF* *V600E* mutation patients remains undefined. In patients with unresectable stage IIIA NSCLC, consolidation immunotherapy after concurrent chemoradiotherapy (the PACIFIC regimen) confers a survival benefit in the population with PD-L1 expression ≥1% (29). The PACIFIC study selectively excluded individuals with *EGFR* or *ALK* driver alterations, but not those harboring *BRAF V600E*; therefore, patients with *BRAF* mutations may also derive clinical benefit from this treatment strategy. Nevertheless, this approach inevitably entails the toxicities of thoracic radiotherapy and platinum-based chemotherapy, along with a heightened risk of interstitial pneumonitis during subsequent immune checkpoint blockade. Previous studies have suggested that patients with high PD-L1 expression are more likely to have primary resistance to Epidermal Growth Factor Receptor Tyrosine Kinase Inhibitor (EGFR-TKI) treatment, which may be related to the suppressive tumor microenvironment (30–32). A retrospective study (*n* = 205) found that high PD-L1 expression (≥50%) was associated with shorter first-line time to treatment failure (TTF) in patients with advanced NSCLC harboring *BRAF V600* mutation, regardless of targeted therapy (BRAF inhibitor/MEK inhibitor) or immunotherapy, underscoring the distinct immune microenvironment biology of this molecular subset (33). Furthermore, due to the extremely low incidence of dMMR/MSI-H in lung cancer, especially in patients with *BRAF V600E* mutations, evidence supporting the predictive value of dMMR/MSI-H for immunotherapy in this population is limited (34, 35). Therefore, the role of PD-L1 and dMMR/MSI-H testing in patients with unresectable stage IIIA NSCLC warrants further investigation. Additionally, for stage IIIA patients with actionable mutations other than EGFR or ALK, the optimal treatment strategy—whether concurrent chemoradiotherapy followed by immunotherapy, concurrent chemoradiotherapy followed by targeted therapy, or neoadjuvant targeted therapy followed by surgery —remains a problem to be solved.

For NSCLC patients without EGFR or ALK alterations, the optimal postoperative treatment remains controversial, even in cases achieving pathological assessment (pCR). In the CheckMate 816 study (36), patients with resectable stage IIB (≥4 cm) to stage IIIA NSCLC who received neoadjuvant immunotherapy and chemotherapy had a 2-year event-free survival (EFS) rate of 85–95% and a 4-year overall survival (OS) rate of up to 95%. These findings suggest that pCR may indicate complete control of systemic disease, as 93% of pCR patients had no detectable mutations in circulating tumor DNA (ctDNA) after surgery. We believe that further adjuvant treatment may not provide additional benefits. However, KEYNOTE-671 (37) and CheckMate-77T (38) studies showed that perioperative immunotherapy significantly reduced the risk of recurrence regardless of whether pCR was achieved, and the overall survival benefit was significant in the entire population. This patient received targeted therapy before the surgery, and the aim of postoperative treatment was to further reduce the risk of recurrence. Options such as adjuvant chemotherapy, adjuvant targeted therapy, adjuvant immunotherapy, or even temporary treatment holidays could be considered. If this patient has the opportunity to undergo PD-L1 testing and ctDNA testing, the results may provide a better therapeutic indication for therapy. For example, PD-L1 expression may indicate immunotherapy, the existence of BRAF V600E in ctDNA may indicate benefits from targeted therapy, the detection of TP53 and other recurrence risk genes may indicate the need for chemotherapy, and a negative ctDNA result may support regular CT reviews. A precise postoperative treatment plan is often based on precise testing.

## Conclusion

This case provides supporting evidence for the perioperative application of BRAF inhibitors and MEK inhibitors in patients with stage IIIA *BRAF V600E* NSCLC, which can improve the prognosis of locally advanced *BRAF V600E NSCLC*.

## Data Availability

The original contributions presented in the study are included in the article/supplementary material, further inquiries can be directed to the corresponding author.

## References

[1] O’Leary CG Andelkovic V Ladwa R Pavlakis N Zhou C Hirsch F . Targeting BRAF mutations in non-small cell lung cancer. Transl Lung Cancer Res. (2019) 8:1119–24. doi: 10.21037/tlcr.2019.10.22 32010589 PMC6976351

[2] Riudavets M Cascetta P Planchard D. Targeting BRAF-mutant non-small cell lung cancer: current status and future directions. Lung Cancer. (2022) 169:102–14. doi: 10.1016/j.lungcan.2022.05.014, 35696864

[3] Guisier F Dubos-Arvis C Viñas F Doubre H Ricordel C Ropert S . Efficacy and safety of anti-PD-1 immunotherapy in patients with advanced NSCLC with BRAF, HER2, or MET mutations or RET translocation: GFPC 01-2018. J Thorac Oncol. (2020) 15:628–36. doi: 10.1016/j.jtho.2019.12.129, 31945494

[4] Negrao MV Skoulidis F Montesion M Schulze K Bara I Shen V . Oncogene-specific differences in tumor mutational burden, PD-L1 expression, and outcomes from immunotherapy in non-small cell lung cancer. J Immunother Cancer. (2021) 9:e002891. doi: 10.1136/jitc-2021-002891, 34376553 PMC8356172

[5] Parisi C Planchard D. BRAF in non-small cell lung cancer: from molecular mechanisms to clinical practice. Cancer. (2025) 131:e35781. doi: 10.1002/cncr.35781, 40172088

[6] Yaeger R Corcoran RB. Targeting alterations in the RAF-MEK pathway. Cancer Discov. (2019) 9:329–41. doi: 10.1158/2159-8290.CD-18-1321, 30770389 PMC6397699

[7] Song H Zhang J Ning L Zhang H Chen D Jiao X . The MEK1/2 inhibitor AZD6244 sensitizes BRAF-mutant thyroid cancer to vemurafenib. Med Sci Monit. (2018) 24:3002–10. doi: 10.12659/MSM.910084, 29737325 PMC5965018

[8] Chappell WH Steelman LS Long JM Kempf RC Abrams SL Franklin RA . Ras/Raf/MEK/ERK and PI3K/PTEN/Akt/mTOR inhibitors: rationale and importance to inhibiting these pathways in human health. Oncotarget. (2011) 2:135–64. doi: 10.18632/oncotarget.240, 21411864 PMC3260807

[9] McCubrey JA Steelman LS Chappell WH Abrams SL Franklin RA Montalto G . Ras/Raf/MEK/ERK and PI3K/PTEN/Akt/mTOR cascade inhibitors: how mutations can result in therapy resistance and how to overcome resistance. Oncotarget. (2012) 3:1068–111. doi: 10.18632/oncotarget.659, 23085539 PMC3717945

[10] Van Allen EM Wagle N Sucker A Treacy DJ Johannessen CM Goetz EM . The genetic landscape of clinical resistance to RAF inhibition in metastatic melanoma. Cancer Discov. (2014) 4:94–109. doi: 10.1158/2159-8290.CD-13-0617, 24265153 PMC3947264

[11] Planchard D Smit EF Groen H Groen HJM Mazieres J Besse B . Dabrafenib plus trametinib in patients with previously untreated BRAF(V600E)-mutant metastatic non-small-cell lung cancer: an open-label, phase 2 trial. Lancet Oncol. (2017) 18:1307–16. doi: 10.1016/S1470-2045(17)30679-4, 28919011

[12] Planchard D Besse B Groen H Groen HJM Hashemi SMS Mazieres J . Phase 2 study of dabrafenib plus trametinib in patients with BRAF V600E-mutant metastatic NSCLC: updated 5-year survival rates and genomic analysis. J Thorac Oncol. (2022) 17:103–15. doi: 10.1016/j.jtho.2021.08.011, 34455067

[13] Odogwu L Mathieu L Blumenthal G Larkins E Goldberg KB Griffin N . FDA approval summary: dabrafenib and trametinib for the treatment of metastatic non-small cell lung cancers harboring BRAF V600E mutations. Oncologist. (2018) 23:740–5. doi: 10.1634/theoncologist.2017-0642, 29438093 PMC6067947

[14] Planchard D Popat S Kerr K Novello S Smit EF Faivre-Finn C . Metastatic non-small cell lung cancer: ESMO clinical practice guidelines for diagnosis, treatment and follow-up. Ann Oncol. (2018) 29:iv192–237. doi: 10.1093/annonc/mdy275, 30285222

[15] Giustini NP Jeong AR Buturla J Bazhenova L. Advances in treatment of locally advanced or metastatic non-small cell lung Cancer: targeted therapy. Clin Chest Med. (2020) 41:223–35. doi: 10.1016/j.ccm.2020.02.003, 32402358

[16] Long GV Hauschild A Santinami M Atkinson V Mandalà M Chiarion-Sileni V . Adjuvant dabrafenib plus trametinib in stage III BRAF-mutated melanoma. N Engl J Med. (2017) 377:1813–23., 28891408 10.1056/NEJMoa1708539

[17] Dummer R Ascierto PA Gogas HJ Arance A Mandala M Liszkay G . Encorafenib plus binimetinib versus vemurafenib or encorafenib in patients with BRAF-mutant melanoma (COLUMBUS): a multicentre, open-label, randomised phase 3 trial. Lancet Oncol. (2018) 19:603–15. doi: 10.1016/S1470-2045(18)30142-6, 29573941

[18] Marchetti A Felicioni L Malatesta S Grazia Sciarrotta M Guetti L Chella A . Clinical features and outcome of patients with non-small-cell lung cancer harboring BRAF mutations. J Clin Oncol. (2011) 29:3574–9. doi: 10.1200/JCO.2011.35.9638, 21825258

[19] Jordan EJ Kim HR Arcila ME Barron D Chakravarty D Gao JJ . Prospective comprehensive molecular characterization of lung adenocarcinomas for efficient patient matching to approved and emerging therapies. Cancer Discov. (2017) 7:596–609. doi: 10.1158/2159-8290.CD-16-1337, 28336552 PMC5482929

[20] Tabbò F Pisano C Mazieres J Mezquita L Nadal E Planchard D . How far we have come targeting BRAF-mutant non-small cell lung cancer (NSCLC). Cancer Treat Rev. (2022) 103:102335. doi: 10.1016/j.ctrv.2021.102335, 35033867

[21] Umemura S Kuo CH Sriuranpong V Thiagarajan M Akewanlop C Murata Y . Clinical impact of the rapid genomic screening platform for non-small cell lung cancer in Asia-Pacific (LC-SCRUM-AP). J Clin Oncol. (2024) 43:Abstract 8637. doi: 10.1200/JCO.2024.42.16_suppl.8637

[22] Chaft JE Swanson S Lee JM Lin J Tsukada H Li T . Preliminary results of the lung Cancer mutation consortium LCMC4 evaluation of actionable drivers in early stage lung cancer (LEADER) screening. J Clin Oncol. (2024) 42:Abstract 8068. doi: 10.1200/JCO.2024.42.16_suppl.8068

[23] Hauschild A Dummer R Santinami M Atkinson V Mandala M Merelli B . Long-term follow up for adjuvant dabrafenib plus trametinib in stage III BRAF-mutated melanoma: final results of the COMBI-AD study. J Clin Oncol. (2024) 42:9500-9500. doi: 10.1056/NEJMoa1708539

[24] Wang JR Zafereo ME Dadu R Ferrarotto R Busaidy NL Lu C . Complete surgical resection following neoadjuvant dabrafenib plus trametinib in BRAF(V600E)-mutated anaplastic thyroid carcinoma. Thyroid. (2019) 29:1036–43. doi: 10.1089/thy.2019.0133, 31319771 PMC6707029

[25] Lee JM Toloza EM Pass HI Johnson BE Heymach JV Sholl L . P2. 01-06 NAUTIKA1 study: preliminary efficacy and safety data with neoadjuvant alectinib in patients with stage IB-III ALK+ NSCLC. J Thorac Oncol. (2023) 18:2. doi: 10.1016/j.jtho.2023.09.511

[26] Liu C Lu M Yang Y Wang X Ma F Liu X. Case report: major pathologic response induced by neoadjuvant treatment using BRAF and MEK inhibitors in a patient with stage IIIA lung adenocarcinoma harboring BRAF V600E-mutation. Front Oncol. (2022) 12:961539. doi: 10.3389/fonc.2022.961539, 36003777 PMC9393753

[27] Huang Z Wang Y Li B Xu Y Huang G Song Y . Neoadjuvant BRAF and MEK inhibitor therapy elicits pathological complete response in stage IIIA non-small cell lung cancer harboring BRAF V600E mutation: a case report. Thorac Cancer. (2024) 15:1825–8. doi: 10.1111/1759-7714.15409, 39020500 PMC11333294

[28] Dudnik E Peled N Nechushtan H Wollner M Onn A Agbarya A . BRAF mutant lung Cancer: programmed death ligand 1 expression, tumor mutational burden, microsatellite instability status, and response to immune check-point inhibitors. J Thorac Oncol. (2018) 13:1128–37. doi: 10.1016/j.jtho.2018.04.024, 29723688

[29] Spigel DR Faivre-Finn C Gray JE Vicente D Planchard D Paz-Ares L . Five-year survival outcomes from the PACIFIC trial: durvalumab after chemoradiotherapy in stage III non-small-cell lung cancer. J Clin Oncol. (2022) 40:1301–11. doi: 10.1200/JCO.21.01308, 35108059 PMC9015199

[30] Yang CY Liao WY Ho CC Chen KY Tsai TH Hsu CL . Association between programmed death-ligand 1 expression, immune microenvironments, and clinical outcomes in epidermal growth factor receptor mutant lung adenocarcinoma patients treated with tyrosine kinase inhibitors. Eur J Cancer. (2020) 124:110–22. doi: 10.1016/j.ejca.2019.10.019, 31760310

[31] Su S Dong ZY Xie Z Yan LX Li YF Su J . Strong programmed death ligand 1 expression predicts poor response and De novo resistance to EGFR tyrosine kinase inhibitors among NSCLC patients with EGFR mutation. J Thorac Oncol. (2018) 13:1668–75. doi: 10.1016/j.jtho.2018.07.016, 30056164

[32] Hsu KH Huang YH Tseng JS Chen KC Ku WH Su KY . High PD-L1 expression correlates with primary resistance to EGFR-TKIs in treatment naïve advanced EGFR-mutant lung adenocarcinoma patients. Lung Cancer. (2019) 127:37–43. doi: 10.1016/j.lungcan.2018.11.021, 30642549

[33] Wiesweg M Alaffas A Rasokat A Saalfeld FC Rost M Assmann C . Treatment sequences in BRAF-V600-mutated NSCLC: first-line targeted therapy versus first-line (chemo-) immunotherapy. J Thorac Oncol. (2025) 20:1328–35. doi: 10.1016/j.jtho.2025.04.016, 40345491

[34] Ea L. Higher predilection of driver mutation positive squamous lung cancer in the Asia Pacific region. J Thorac Oncol. (2025) 20:S98–S120. doi: 10.1016/s1556-0864(25)00595-7

[35] Ambrosini M Manca P Nasca V Sciortino C Ghelardi F Seligmann JF . Epidemiology, pathogenesis, biology and evolving management of MSI-H/dMMR cancers. Nat Rev Clin Oncol. (2025) 22:385–407. doi: 10.1038/s41571-025-01015-z, 40181086

[36] Provencio M Serna-Blasco R Nadal E Insa A García-Campelo MR Casal Rubio J . Overall survival and biomarker analysis of neoadjuvant Nivolumab plus chemotherapy in operable stage IIIA non-small-cell lung Cancer (NADIM phase II trial). J Clin Oncol. (2022) 40:2924–33. doi: 10.1200/JCO.21.02660, 35576508 PMC9426809

[37] Spicer JD Garassino MC Wakelee H Liberman M Kato T Tsuboi M . Neoadjuvant pembrolizumab plus chemotherapy followed by adjuvant pembrolizumab compared with neoadjuvant chemotherapy alone in patients with early-stage non-small-cell lung cancer (KEYNOTE-671): a randomised, double-blind, placebo-controlled, phase 3 trial. Lancet. (2024) 404:1240–52. doi: 10.1016/S0140-6736(24)01756-2, 39288781 PMC11512588

[38] Provencio M Awad MM Spicer J Janssens A Moiseenko FV Gao Y . Clinical outcomes with perioperative nivolumab (NIVO) by nodal status among patients (pts) with stage III resectable NSCLC: results from the phase 3 CheckMate 77T study. J Clin Oncol. (2024) 42:LBA8007-LBA8007. doi: 10.1200/jco.2024.42.17_suppl.lba8007

